# Developing Digital Mental Health Tools With Culturally Diverse Parents and Young People: Qualitative User-Centered Design Study

**DOI:** 10.2196/65163

**Published:** 2025-04-22

**Authors:** Isobel Butorac, Roisin McNaney, Joshua Paolo Seguin, Patrick Olivier, Jaimie C Northam, Lucy A Tully, Talia Carl, Adrian Carter

**Affiliations:** 1 School of Psychological Sciences Monash University Clayton Australia; 2 School of Computing and Information Systems The University of Melbourne Melbourne Australia; 3 Action Lab Faculty of Information Technology Melbourne Australia; 4 The School of Psychology Faculty of Science The University of Sydney Camperdown Australia; 5 Growing Minds Australia Australia's Clinical Trials Network in Child and Youth Mental Health Sydney Australia

**Keywords:** digital mental health, young people, cultural diversity, web-based and mobile health interventions, qualitative methods, user-centered design, human-computer interaction

## Abstract

**Background:**

Approximately 39% of young people (aged 16-24 y) experience mental ill health, but only 23% seek professional help. Early intervention is essential for reducing the impacts of mental illness, but young people, particularly those from culturally diverse communities, report experiencing shame and stigma, which can deter them from engaging with face-to-face services. Digital mental health (DMH) tools promise to increase access, but there is a lack of literature exploring the suitability of DMH tools for culturally diverse populations.

**Objective:**

The project was conducted in partnership with a large-scale national DMH organization that promotes evidence-based early intervention, treatment, and support of mental health in young people and their families. The organization wanted to develop a self-directed web-based platform for parents and young people that integrates psychological assessments and intervention pathways via a web-based “check-in” tool. Our project explored the views of culturally diverse parents and young people on the opportunities and barriers to engagement with a web-based DMH screening tool.

**Methods:**

We conducted a 2-phase qualitative study aiming to identify potential issues faced by culturally diverse communities when engaging with DMH tools designed for the Australian public. We worked with 18 culturally diverse participants (parents: n=8, 44%; young people: n=10, 56%) in a series of design-led workshops drawing on methods from speculative design and user experience to understand the opportunities and barriers that organizations might face when implementing population-level DMH tools with culturally diverse communities. NVivo was used to conduct thematic analyses of the audio-recorded and transcribed workshop data.

**Results:**

Five themes were constructed from the workshops: (1) trust in the use and application of a DMH tool, (2) data management and sharing, (3) sociocultural influences on mental health, (4) generational differences in mental health and digital literacy, and (5) stigma and culturally based discrimination in mental health support.

**Conclusions:**

The emergent themes have important considerations for researchers wishing to develop more inclusive DMH tools. The study found that healthy parent-child relationships will increase engagement in mental health support for young persons from culturally diverse backgrounds. Barriers to engagement with DMH tools included culturally based discrimination, the influence of culture on mental health support, and the potential impact of a diagnostic label on help seeking. The study’s findings suggest a need for culturally safe psychoeducation for culturally diverse end users that fosters self-determination with tailored resources. They also highlight important key challenges when working with culturally diverse populations.

## Introduction

### Background

Approximately 13% of the global population living with mental health problems are young people (aged 16-24 y) [1]. Young people with mental health disorders also experience higher rates of morbidity and mortality risk than the general population, leading to a 10- to 20-year reduction in life expectancy [2,3]. These rates were compounded during the COVID-19 pandemic, during which globally 1 in 4 young people experienced clinically elevated levels of depression, and 1 in 5 experienced clinically elevated levels of anxiety [4]. Nevertheless, during this period, there was no concurrent increase in the uptake of mental health support [5]. For young people, untreated mental health problems can have long-lasting impacts due to the important social, emotional, and cognitive developmental changes that are being experienced simultaneously [6].

### Cultural Diversity in the Context of Australia

Australia is a culturally diverse society, with 27.6% of its population born overseas [1]. This diversity spans across differences in cultural identity, language, country of birth, religion, heritage, and national origin [7]. Shaped by a legacy of colonialism, racial inequality has influenced Australia and its relationship with racial and ethnic minority immigrants [8] and First Nations peoples [9].

Studies have identified higher rates of mental ill-health among culturally diverse groups compared to the broader Australian population [10,11]. This is, in part, due to mental health risk factors, such as language barriers, cultural adjustment, the loss of family connection, and an inability to apply knowledge and occupational skills to attain meaningful employment, all of which hinder active participation in society for people from culturally diverse backgrounds [12]. Young people from culturally diverse backgrounds tend to be the most reluctant to seek help, and if they do, they typically turn to informal sources such as family, friends, and elders [13,14]. Among those who have sought professional support, many report that the types of support they were offered were inaccessible, culturally inappropriate, or lacking cultural relevance [15-17].

Common barriers to mental health care for culturally diverse communities include a lack of information and knowledge about available services, language differences, culturally specific conceptions of mental health, and lengthy waitlists [18-22]. Discrimination can be experienced throughout a person’s mental health journey, with studies showing that culturally diverse people are far less likely to engage in mental health visits or seek specialist care than White ethnic groups [23]. Research has also found that clinicians are less likely to involve culturally diverse patients in friendly discussions or include them in the treatment decision-making process [24]. Instead, people from culturally diverse backgrounds tend to access mental health services through clinical emergency services during an acute crisis rather than accessing early interventions before the problem is severe, hindering their prospect of recovery [25,26]. People from culturally diverse backgrounds in Australia also experience more involuntary hospital admissions for mental health treatment [16].

### Current Landscape of Digital Mental Health Tools and Opportunities

Digital mental health (DMH) tools (eg, apps and web-based services) delivered through smartphones [27-31], computers [32,33], and wearable devices [34,35] hold promise for overcoming many of the aforementioned barriers [36]. DMH tools can include self-directed online interventions [37,38], mental health screenings or “check-ins” to assess mental health status [39], digital health apps that allow the real-time monitoring of mental health [40,41], chatbots to support help seeking [42], and even interventions on specialist technologies such as virtual reality headsets [43,44]. Studies have shown that young people and parents prefer digital treatment over face-to-face care [45,46]. DMH tools offer easily accessible, low-cost services to address common mental health problems [47,48]. Self-directed DMH tools may offer alternative or complementary options to face-to-face clinical care, with the potential to provide support to people on waitlists for clinical care in remote locations or those who may not have the financial means to seek face-to-face treatment [43]. There is increasing interest in developing culturally sensitive DMH tools [17,49], with some DMH apps and online platforms being designed especially for ethnically diverse groups [50]. However, such examples remain limited; moreover, considerations of diversity, equity, and inclusion are rarely integrated into their design or evaluation [51].

Online self-screening tools provide useful information about self-reported mental health symptoms, offering clinical cutoffs to indicate when further assessment or support is needed, along with relevant resources for support. They also allow anonymous pathways for disclosing mental health symptoms and offer digital interventions [4,52,53] that have been shown to be fundamental for the mental health journey of culturally diverse users because digital interventions and resources are more likely to reach culturally diverse populations who may not have access to or are not yet engaging in mental health support [54]. Large-scale DMH platforms that incorporate screening, access to online clinicians, self-directed resources, and curated suites of trusted apps are increasingly being adopted by public health organizations across Western contexts (eg, the UK National Health Service’s Talking Therapies program) [48]. However, while these public-facing DMH tools are intended for use by all of society, they tend to be designed by and for the dominant Western culture [55].

In addition, DMH tools hold the potential for supporting mental health beyond traditional 1-to-1 contexts. Online health-oriented communities offer benefits such as peer support networks (eg, Headspace and Orygen both offer online youth mental health support in Australia). Similarly, social media groups focused on mental health have been shown to play a substantial role for end users in rural areas, where geographic location limits access to mental health service providers [56]. These groups provide support for guided online therapy [57], skills building [58], symptom monitoring [59], and social connection [60,61]. However, among culturally diverse communities, where English may be a second language, willingness to engage in these types of peer support offerings tends to be lower [50].

### Designing With Diverse Populations

There is increasing recognition of the importance of designing digital services tailored for culturally diverse populations using responsive and inclusive design processes. Many researchers and designers in this space have adopted participatory research and user-centered approaches wherein members of the community are involved in the design process to identify their unique needs, goals, and concerns and to ensure that these are considered and integrated into the final service design [62-67]. Technologies developed for an underserved community are most impactful when designed with members of that community [68]. The use of user-centered approaches has been shown to improve the acceptability, effectiveness, and contextual relevance of novel technologies for communities [55]. Engaging members of the community in a design process often requires creative methods and tools to ensure that it is safe, beneficial, and easy for them to participate. Some studies have used creative tool kits, personas, and vignettes to scaffold design thinking [69,70].

User-centered design approaches that recommend the inclusion of meaningful engagement with consumers and carers in the service design are also increasingly recognized among mental health researchers and practitioners, including government agencies [71]. Research interest in culturally sensitive design through participatory research and user-centered design methods is mounting [72-74]. However, despite the political and cultural shift, there is still only a small, though growing, body of work in the field of human-computer interaction that explores the needs of culturally diverse populations in the area of DMH services [17,49,75,76]. While these studies provide useful insights, they tend to focus on a single community or culture and offer specific rather than generalized interventions. While there are steps being taken to engage culturally diverse communities in technology design across various fields of research, there are still various mental health factors that could impact the uptake of novel DMH tools (eg, cultural concepts of mental health and stigma around mental health). As such, it is vital that we aim to better understand how to design tools that account for these factors and enhance service engagement for culturally diverse communities. For the successful implementation of clinical innovations at a public scale, novel digital solutions need to be designed *with* diverse populations to ensure that these solutions meet their needs [17,77]. Engaging with the perspectives of groups considered marginalized and those outside Western traditional norms will help ensure that DMH tools are designed more inclusively [78,79].

Building on these insights, we worked with culturally diverse young people and parents to design a national digital self-directed mental health check-in tool, called Growing Minds Check-In (GMCI), for the Australian public. The GMCI aims to provide an assessment of mental health and well-being symptoms as well as recommendations to tailored online mental health services and information. This study engaged with user-centered design due to the restrictions on the level of involvement the participants would have on the final production of the GMCI. Due to the narrow development timelines, the participants in this study were able to contribute to the design process but did not have decision-making powers; instead, they were involved in appropriate ways throughout the development of the GMCI [80]. We conducted a 2-phase study aiming to identify potential issues faced by culturally diverse communities when engaging with DMH tools such as the GMCI. The purpose of this study is to describe the attitudes, barriers, and benefits associated with DMH tool engagement among culturally diverse parents and young people. This information is likely to be relevant to DMH interventions for parenting and child well-being; it may also be relevant to other online programs, especially those delivered as universal public health interventions. We propose a set of design strategies for developing public-facing DMH tools and provide reflections on our own design process and the challenges we faced while engaging with culturally diverse communities in research.

## Methods

### Study Context

This project was conducted in partnership with the Growing Minds Australia Clinical Trials Network, Australia’s first clinical trials network for child and youth mental health research [81]. It is part of the first phase of design for the Growing Minds Australia Clinical Trials Network flagship trial, the GMCI: a national online platform for parents and young people that integrates a mental health and well-being “check-in” with personalized feedback and suggestions for online mental health services and resources. We came into the project with the aim of focusing inquiry on how the future-facing platform could be designed more inclusively to ensure that it is relevant and accessible for diverse communities. We used a speculative DMH app containing features similar to those proposed by digital phenotyping software that could collect both passive and active data.

**Figure 1:**
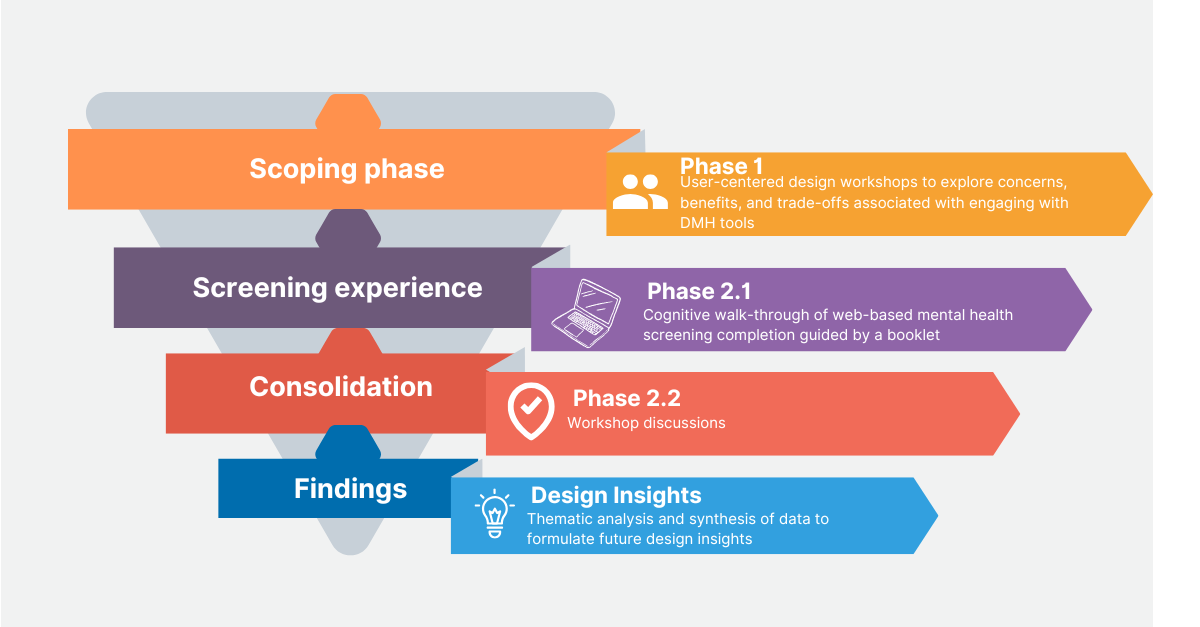
Visualization of the study design. DMH: digital mental health.

The study comprised 2 phases to engage groups of parents and young people from culturally diverse backgrounds with lived experience of mental health challenges. The first was a scoping phase that applied user-centered design methods, which included using speculative DMH tools to tease out potential benefits, concerns, and trade-offs. The second was a prototype exploration phase, whereby a proposed nationwide mental health screening tool was used as an artifact for participants to determine possible challenges and ethical issues that culturally diverse populations may face when engaging with DMH tools. This included a facilitated emotional walk-through of the screening tool using a workbook, followed by a data consolidation workshop where participants reflected on their experiences of using the tool. We aimed to identify the potential benefits and possible challenges of using a large-scale web-based check-in service and incorporate these insights into a more inclusive design for future DMH technologies. The design justice framework was applied to the design of the research questions (RQs), the activity schedule, and the analysis of the findings. This approach promotes the exploration of how benefits and burdens are distributed across groups, challenging design-driven inequalities by using an intersectional lens that considers race, gender, sexual orientation, and culture [82,83]. This framework incorporates participatory and user-centered design methods that prioritize community needs and encourage researchers to consider how power can shape participation in the design processes [82] (Figure 1).

### Ethical Considerations

Our study received ethics approval from Monash University (31138). All participants were provided with an explanatory statement detailing the project and were asked to read and sign a consent form before participating. These documents were provided to participants in advance of the workshops. Consent and demographic information (eg, country of origin and culture they identify with, years lived in Australia, and languages spoken at home) were collected before the workshops began. Participants across all study phases were compensated Aus $40 (US $24) per hour for their time. Data was anonymized with all names removed from the data. Names were changed during transcription before any analysis commenced.

### Recruitment

We partnered with a multicultural community mental health organization to recruit participants and facilitate the workshops. We used the organization’s advertising channels to recruit participants who were involved as volunteers with the organization and were likely to be mental health literate. Workshop participants were required to have a high school level of spoken English due to time limitations and budget restrictions, which precluded the use of translators. Calls for participation in the workshops were advertised through flyers distributed via the community organization. For inclusion in the study, participants needed to identify as either having lived experience with mental health problems or having children who had experienced mental health challenges. These workshops were conducted in person and therefore limited to communities living in Victoria, Australia. A majority of the participants (12/18, 67%) were from South or East Asia, reflecting the demographic composition of the Australian population [1]. In phase 2, we included an Anglo-Australian participant (P8) who was recommended as a research participant by the mental health organization. Although not from a culturally diverse background, this participant was an experienced peer support worker with multicultural populations, had lived experience of complex mental health challenges, and was a parent. Phase 1 and phase 2 participant demographics are presented in Table 1.

**Table 1:** Phase 1 and phase 2 participant demographics.

Phases and participant IDs		Age (y)	Parent or young person	Duration of residence in Australia (y)	Self-identified cultural background	Languages spoken at home
**Phase 1**						
	P1	65	Parent	35	Indian	English, Tamil, and Hindi
	P2	57	Parent	35	Indian	English and Tamil
	P3	53	Parent	23	Indian	Tamil
	P4	67	Parent	50	German	German and English
	P5	48	Parent	48	Italian	Italian and English
	P6	58	Parent	41	Indian	Hindi, Punjabi, and English
	P7	67	Parent	67	Australian and German	English and German
	YP1	22	Young person	3.5	Turkish	Turkish
	YP2	16	Young person	7	German and Indian	German and English
	YP3	22	Young person	11	Malaysian	Cantonese and English
	YP4	21	Young person	4	Indian	Hindi
	YP5	23	Young person	21	Han Chinese	Mandarin Chinese
	YP6	22	Young person	12	Australian Chinese	Mandarin Chinese
	YP7	18	Young person	17	Malaysian	English
	YP8	24	Young person	5	South African	Zulu
	YP9	24	Young person	11	Malaysian Chinese	Cantonese, Mandarin, and Bahasa Melayu
	YP10	21	Young person	20	Indian or Hindu	English and Tamil
**Phase 2**						
	P1	65	Parent	35	Indian	English, Tamil, and Hindi
	P2	57	Parent	35	Indian	English and Tamil
	P3	53	Parent	23	India	Tamil
	YP3	22	Young person	11	Malaysian	Cantonese and English
	YP4	21	Young person	4	India	Hindi
	YP10	21	Young person	20	India or Hindu	English and Tamil
	P7	67	Parent	67	Australian and German	English and German
	P8	47	Parent	47	Australian	English

### Phase 1: Contextual Inquiry Through User-Centered Design

#### Overview

We conducted 2 user-centered design workshops: one with 10 young people (female: n=7, 70%; male: n=3, 30%; aged 16-25 y) and another with 8 parents of young people (female: n=4, 50%; male: n=4, 50%; aged 21-67 y). Each workshop included a researcher facilitator and a cofacilitator provided by the mental health organization who assisted with recruitment. Power imbalances were mitigated by conducting the workshops at an office space provided by the multicultural organization, where participants were affiliated and felt comfortable. The workshops ran for 3 hours with a 20-minute refreshment break. We examined 3 broad RQs:

1. How do culturally diverse end users perceive the value of mental health data? With whom would they share their mental health data? How might their mental health data differ in the future?
2. How is screening information communicated and understood? What impact would this have on therapeutic relationships?
3. What are the social factors that influence the likely use and application of DMH technologies? Are culturally diverse users concerned about third-party use of their data?

We aimed to not only explore the inclusion of culturally diverse communities in the design of a digital platform but also consider the impact of future data use, particularly how an end user’s sensitive data may later be used. Given the propensity for alternative data use by third parties, we wanted to better understand the degree to which culturally diverse end users worried about how their data may be used and whether they felt it would have future implications.

**Figure 2:**
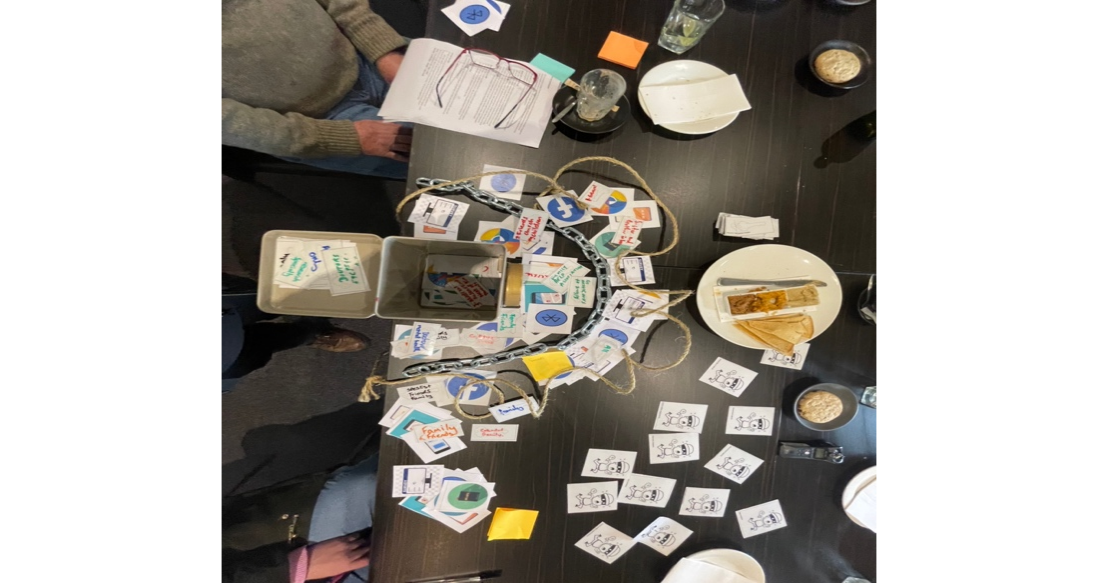
The “digital vault” activity in phase 1.

The workshop sessions followed a semistructured activity guide that was developed by the authors and reviewed by colleagues independent of the study (refer to Multimedia Appendix 1 for details). We began each session with an icebreaker activity and initiated a group discussion to set the context for the following activities. Using a whiteboard and markers, we asked participants about their understanding of mental health and what makes for good and poor mental health. This activity was intended to provide insight into the participants’ mental health literacy. We then provided participants with a speculative design probe, the “mental health vault” (Figure 2), to unpack abstract conceptions of sensitive digital health data. The vault included 3 layers to indicate varying levels of personal information that may be uncovered by DMH tools: (1) a locked safe represented by a metal box (most personal), (2) an inner layer bound by a thick metal chain, and (3) an outer layer framed by a thin rope (least personal). The activity schedule included four activities: (1) digital vault, (2) blank keys, (3) data sharing, and (4) future forecasting. These activities were centered on an imagined DMH tool that could actively and passively collect end user data, which differs from the GMCI’s intended use and functionality as a web-based tool that provides personalized feedback and suggestions for online mental health services and information matched to need.

#### Activity 1: Digital Vault

The aim of this activity was to address RQ 1 by gauging participants’ level of understanding of mental health data, how sensitive they perceived the data to be, and with whom they would want to share the data. To scaffold their thinking, we provided stimuli picture cards of potential mental health data points (eg, a diary, a mobile phone, GPS data, survey test results, and social media data) and asked participants to place each card, depending on how secure they felt each data point was, within the different layers of the vault (ie, inside the metal box, within the chain, or in the outer rope layer). The most private data points were placed inside the metal box, while the least private were positioned in the outer rope layer. After participants arranged the cards according to their preferences, we asked them to describe what each layer represented to them and why they placed each data point where they did (eg, why they would be okay with sharing social media data publicly but not their survey test results).

#### Activity 2: Blank Keys

The aim of this activity was to understand whom the participants were willing to share their data with and their reasons, addressing RQ 1. Using an inductive approach, we used this component of the activity to leverage and broaden the discussion to generate ideas on new “trusted” or “nontrusted” people that the participants could think of on their own, without the researcher’s prompts or suggestions. We gave participants 6 blank paper “keys” and asked them to write down the individuals (eg, their imam, family member, or teacher) to whom they would be willing to grant access to the vault. They then placed each key in the vault layer they felt most comfortable allowing that individual to access. Next, we asked participants to use these blank keys to write down who they thought might “rob” the vault and to place the newly labeled keys in the layer of the vault they felt the “data robber” might access. The activity schedule script included the following prompt: “Now let’s imagine digital bank robbers broke into your vault. What is the worst thing that can happen? Who might the robbers be? Why would they want your data? Which specific area of the vault would they be most interested in?”

#### Activity 3: Data Sharing

Building on the digital vault scenario, we introduced a “digital vault manager” who has access to the data contained in the digital vault and could use the data to identify mental health issues. This activity addressed RQ 2 and RQ 3. We then asked the participants whether they would want anyone to know about mental health challenge identified, and if so, whom they would share it with. This activity was designed to get participants thinking about their individual perspectives as well as how others may feel about having their personal data shared with the intention of helping individuals or their community (eg, whether a school or current employer should be notified).

#### Activity 4: Future Forecasting

This activity examined how screening information is likely to be communicated, understood, and applied in face-to-face sessions with a clinician. This activity addressed RQ2 and RQ3. We asked participants to imagine that the digital vault had been passively collecting data and had generated a prediction about their mental health over the next 20 years. First, we gathered emotional reactions to the prediction using printed emoji faces and by asking participants to indicate how they felt about having predictions made about them. Next, through a discussion-based activity, we explored how the prediction their trust, and sense of empowerment, and agency, as well as how and by whom the prediction would best be communicated. The activity schedule script included the following prompt: “Do you feel empowered knowing this prediction in advance so you can seek help? Do you trust its accuracy? Would you prefer your GP [general practitioner] or mental health clinician had told you? Would you trust it more if your GP told you? Are you concerned about who else is accessing this digital vault from some years ago?”

The findings from the future-facing DMH tool discussion in phase 1 were used to frame the emotional walk-through activity and focus groups with parents and young people in phase 2. We considered the benefits, burdens, and trade-offs that the culturally diverse participants discussed in these workshops and wove them into the emotional walk-through activity in an attempt to highlight challenges with an existing system that could then feed into a future design. This activity series was designed to address RQs 1 to 3.

### Phase 2: Emotional Walk-Through

**Figure 3:**
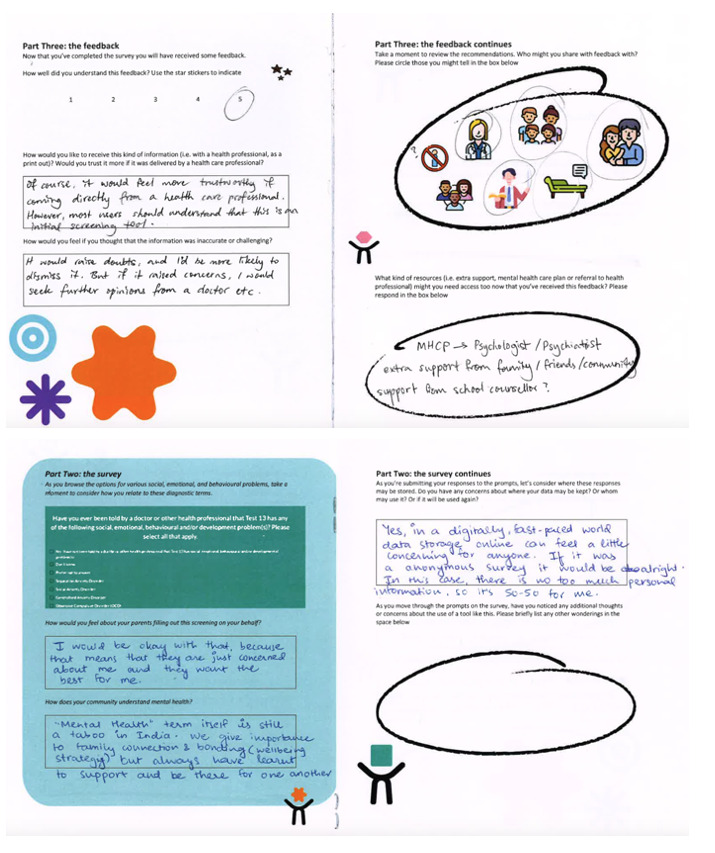
A completed emotional walk-through booklet in phase 2.1.

Using a user experience technique traditionally referred to as a cognitive walk-through, we aimed to explore participants’ emotional responses as they engaged with the interface [84,85]. We adapted a walk-through user testing methodology [86] and created a 6-page emotional walk-through booklet (Figure 3) that was distributed to participants along with a link to a prototype of the web-based mental health screening platform intended for national deployment in the near future. This was a written task that was designed to help facilitate discussion and ideas about the issues we intended to explore in the prototype testing workshop. Participants (parents and young people) were then invited to take part in a joint consolidation workshop to discuss their thoughts in further detail. We supplied participants with a booklet that was designed by the team, 2 weeks before the workshop to allow them time in their daily schedule to complete the booklet and explore GMCI prototype. Participants used their completed booklets during the session as mnemonic aids to recall their thoughts and experiences. A total of 6 participants (parents: n=3, 50%; young people: n=3, 50%; female: n=3, 50%; male: n=3, 50%) agreed to participate in the joint consolidation workshop. The aim was to elicit their thoughts and feelings about DMH data as they completed the web-based mental health check-in journey. The booklet included a range of questions designed to gauge emotional reactions as well as rating scale assessments as participants used the web-based screening platform. Within each section of the booklet, we included ethical considerations to encourage participants to consider the social impact of the DMH tools, including their understanding of mental health, how their community understands mental health, views on data privacy and management, how they would like to receive feedback about their mental health, and whether they felt they could challenge a mental health assessment. Participants then took part in the consolidation workshop to discuss their experience of using the prototype and to consolidate their ideas and expectations of engaging with an online mental health screening service. The session was broken down into four parts:

1. What are the motivations, benefits, and challenges of accessing such a tool?
2. Where does the data entered on the web go?
3. How well did the participants understand the feedback supplied? Did it resonate with their cultural background?
4. What suggestions did the participants have to make this tool more accepted within specific communities (dos and don’ts).

### Positionality Statement

Our authorship team represents a variety of cultural backgrounds and perspectives on the topic, shaped by our own experiences. We took part in this research because we believe strongly in fostering equal access to mental health services for culturally diverse end users. Our team comprises 8 authors: 5 (62%) female individuals with European, Anglo-Celtic, and Irish heritage; and 3 (38%) male individuals with Anglo-Celtic, Anglo-Australian, and Filipino heritage. Of the 8 authors, 3 (38%) are first-generation migrants to Australia; 4 (50%) identify as parents; and 3 (38%) work with young people, parents, caregivers, and families. Our team consists of clinical and registered psychologists, a neuroscientist and bioethicist, a human-computer interaction and design practitioner, and researchers working in DMH and responsible innovation. We are committed to extending our knowledge and understanding of culturally diverse research practice to empathize and engage with research involving culturally diverse parents and young people in this work. This commitment has informed our approach to the study design and data analysis.

### Data Analysis

Audio recordings from all 3 face-to-face workshops (phase 1: n=2, 67%; phase 2: n=1, 33%) were transcribed and thematically analyzed using NVivo software (Lumivero). An inductive thematic analysis was conducted on all transcripts, without a preexisting coding framework, to identify themes and subthemes [87]. Initial codes were generated by IB to organize the data from phase 1 into potential items of interest. Three additional researchers (AC, RM, and JPS) familiarized themselves with the data from phase 1 and reviewed the initial codes. Iterative coding was then completed by IB, and 21 subthemes were generated that informed the design of the emotional walk-through. Three additional research members (AC, RM, and JPS) cross-coded themes from the emotional walk-through booklets and transcripts from the consolidation workshop. The codes were reviewed to ensure that the themes were clear and descriptive. The coded data from both phases were combined for analysis with 5 themes. In each workshop, an activity schedule was used to scaffold free-flowing discussion among participants. Variations in participant opinions were coded and contrasted to develop the subthemes. Participants were collaborative and brought culturally diverse and unique experiences, offering rich anecdotes in each session.

## Results

Five major themes were developed from the analysis of the data collected from both phases: (1) trust in the use and application of a DMH tool, (2) data management and sharing, (3) sociocultural influences on mental health, (4) generational differences in mental health and digital literacy, and (5) stigma and culturally based discrimination in mental health support.

### The Potential Impact of DMH Tools on Interpersonal and Therapeutic Relationships

The discussions on how DMH tools may impact the therapeutic relationship with a current or future clinician varied; however, both groups (parents and young people) expressed greater trust in a clinician in a face-to-face setting than in a digital tool. Participants in both groups also agreed that they would be more likely to trust a mental health recommendation or diagnosis generated by a DMH tool if a clinician was involved at some point:

I feel like I would still and then like accept whatever the app tells me, but it will be very helpful if the psychiatrist or psychologist are linked with this app. Like they sort of like, acknowledge that this app is useful or something like that.

YP5

Participants acknowledged that they would seek advice from a DMH platform if a clinician was hard to access or as a first step in help seeking. However, some participants (YP10, YP7, YP5, and P2) felt that it was hard to know where to search and stated that they would use DMH tools as a screening tool but would seek help from a trusted health professional, such as a GP, psychiatrist, or psychologist, regarding the information provided by a DMH tools. Conversely, other participants found face-to-face interactions to be “embarrassing” (YP10), “confronting” (P8), or rushed:

I feel like because GPs usually see patients 15 minutes per block, it feels that it’s quite rushed and sometimes GPs wouldn’t take the time to go over time...it will make us feel that we’re not as like important.

YP9

Once a recommendation or treatment suggestion has been provided by DMH tools, participants in both groups (P6, YP5, and P3) expressed a desire to process it on their own first:

I would want to see the information first and then several steps down the road, consulting with a specialist might be an option.

P3

Once they had reached a point of acceptance regarding the recommendation or treatment suggestion, there was variability in whom they would share the data with, with disclosures to family, friends, and practitioners varying within both groups. Some parents (P1, P2, and P3) preferred to be given the recommendation before sharing it with their children. Two younger participants (YP2 and YP5) were happy to share the information with their parents and siblings, while many of the other young participants (YP1, YP3, YP8, YP9, and YP10) indicated that they would prefer to share it with a trusted contact or friend—rather than with their parents and siblings—who they believed would help put them in touch with a professional or assist in accessing additional resources.

One young person (YP3) suggested having the option to choose to download feedback from a DMH tool depending on the severity of the user’s mental health problems. Another young person made the following suggestion:

If you delete it, they ask you, “Are you sure?” or like, “Are like are you too busy?” “Is it distracting?” Like it ask you the reasons why you are downloading it. Have you met your match? [similar to a dating app] I think the same should be with the app. Like if you don’t like the app, but you still, you downloaded this, but you still feel like you need someone to talk to, call these numbers. And it shouldn’t be triple zero, because why would I call triple zero in my, like I don’t...maybe triple zero can be like the last thing, but like call this person or locate the nearest center or psychologist, or locate your nearest person or something else.

YP8

Suggestions were made in regard to having a trusted contact who would receive a notification if a user was determined to be at high risk for poor mental health, with the understanding that the trusted person would not be obligated to assume responsibility. Two parents (P7 and P8) emphasized the importance of a DMH tool’s recommendation or resource that offered access to peer support networks. Another suggestion was for the user to predetermine a lower threshold for a risk notification if they were at risk of increased suicidality, allowing earlier intervention:

There’s blurred lines to it, but at least like...having an accountability system...accepting, declining, being able to be there, or if I’m not, if I can be an emergency contact, but I’ll accept, just going through this with you. Like, being there with you but I will not be your emergency contact. Like, I can share experience. In the case where it is an emergency, I don’t want to be an emergency contact, but I can maybe assist you in contacting your emergency contact or something.

YP8

Participants agreed that DMH tools should offer additional resources and alternative options once a screening or check-in is initiated, such as “resources to learn more about what I might be going through” (YP4). Both parents and young people emphasized that healthy parent-to-parent and child-to-parent relationships were important in cases where parents chose to complete a screening on behalf of their child. A parent elaborated as follows:

Obviously we agree to disagree on a lot of topics, but you know, after a certain years, you know your child very well. And...it depends on the relationship between the father and the mother as well.

P2

The closeness between parent and child would be a significant factor in the quality of input into a DMH tool’s screening process, which would then impact the meaningfulness of any subsequent recommendation or prediction:

It’d be a case of if you could offer it as a dual package. So if the child was up to do it with their parents as one option, then you’d have, so you’d have three options, you’d have an adult, you’d have the youth separate, and then you’d have a child and a youth and a parent combo...there’s something to suit everyone depending on their communication space with their family members and how open they are to their mental health.

P10

DMH tools were suggested as a complementary support alongside a clinician in both phase 1 workshops. Additional benefits of using DMH tools included setting reminders or nudges to promote healthy activities such as going for a walk or engaging in mindfulness meditation. A participant in the workshop for young persons also suggested that DMH tools could be useful for users with acute mental health challenges:

I think it would be good for bipolar disorder. For example, if you were able to get a new notification about your hyper episode, then you would able, you wouldn’t be able to change your episodes like change your, arrange your calendar according to that.

YP1

In relation to the technology, a young person described enjoying how music apps and YouTube catered to her mood:

So I relate how I’m feeling, even if it’s like a chronic like mental illness, like how I’m feeling is through my algorithm of my music. So like I’d be on YouTube and I’d probably right now I’m, like...I just figured out I had ADHD so like I’m like constantly watching things that have to do with that. So like I feel like different platforms can algorithmize my personal experience.

YP8

Another young person echoed this and made the following suggestion:

I think technology in essence should be supporting humans and human work, rather than trying to replace it. Um, if my GP or my psychologist fails to diagnose or intervene with me early, I recognize that everything they know about me is what I’ve told them, and those people are human. It’s people they might miss things. So I’ll be more than happy for this sort of data to assist in diagnosing me. Um, and together it will be more valid that just one source or the other.

YP7

In summary, when considering how results are communicated and what impact DMH tools may have on clinical practice, culturally diverse end users wanted to first process a recommendation or prediction on their own, viewed DMH tools as complementary to face-to-face clinical intervention, and were open to having a trusted contact with whom a recommendation or prediction could be shared.

### Data Management and Sharing

Conversations in both phases established that participants had a moderate level of understanding about their personal data and how to manage the data. They acknowledged being aware of daily data collection via internet searches and app use and that the data can be outsourced to different countries and third parties through data harvesting (P3, YP10, P2, P7, YP7, YP8, and YP5). A participant in the workshop for young persons suggested that some data have to be shared to build more accurate and personalized apps:

I feel like data collection should be a two-way street. If you, I mean, like it should be mutual benefit, right?

YP7

Unauthorized data access by governments, concerns after experiences with surveillance measures during the COVID-19 pandemic, recent government health data breaches, and identity theft were key concerns of participants (P8, P7, P2, YP9, P6, and P5). A participant shared another concern:

It could be mental health related or something else related. And then that information goes to your doctor, you consult a specialist or a GP or someone. And then it goes back to your insurance provider. And the insurance provider knows about it and straight away your premium goes up because they classify you as a high risk.

P3

By contrast, young people were more open to having their data tracked or shared, having grown up with much of their information already available on the web and accessible by others. However, a young person acknowledged the difficult dilemma of accepting data collection in exchange for a more personalized online experience but described it as a “gray area” when it comes to broader data collection, stating as follows:

When they click “Accept All,” that gives a warrant basically, and that’s nothing that you can do because the website can just say, “You said, ‘Accept All’” and continue. That’s the warrant for me to say...to collect all your data and personalize it for you. And I feel like it’s, it’s quite a grey area basically.

YP7

Another young person expressed uncertainty regarding their preference for certain data points to be collected or tracked by specific services:

I would want my location to be tracked by medical services. Say, for example, I get hurt and then, um, my phone...like my watch has this thing where it says, “Accident Detected.” But I think about who gets access to what data and what they do with it and I think that’s also really hard to track so it depends.

YP2

In summary, culturally diverse mental health service users valued data about mental health, with variation in openness to data sharing and how the data may be shared in future.

### Sociocultural Influences on Mental Health

Participants’ experiences with mental health were grounded in sociocultural complexities, with variations in cultural understandings of mental health, help seeking, parental influence, community and spiritual support, and migration status, all playing a role in diverse communities’ engagement with DMH support.

Many of the participants (YP7, YP1, YP8, and P8) self-identified as having lived experience with mental ill-health and discussed the importance of self-understanding when it came to mental well-being. Parents, in particular, recognized the importance of acceptance and education around mental health:

Probably because nobody wants to tell anyone, I’m going through not a very nice day or I’m going through mental health issues, so basically we have to come out of that state so we have to educate ourselves there’s no point in just keeping everything to yourself.

P2

Participants agreed that, outside of the family unit, their ethnic communities placed more importance on family connection and bonding than mental health specifically. This was highlighted in the comment written by a young person in the emotional walk-through booklet:

The “Mental health” term itself was still a taboo in India. We give importance to family connection and bonding (as a well-being strategy) but always have learnt to support and be there for one another.

YP4

In addition, some participants (P1, P7, and YP4) discussed how their communities relied on religious leaders for mental health support rather than accessing formal care. However, the dominant cultural lens within a household impacted whether mental health was openly discussed. A parent explained as follows:

I would say it’s very culturally influenced. From my perspective, the culture which we’re connected to, you know. For everything there is a step-up process for you to understand, you know, why this is done, why it is not done. The reasons behind it. So where we grew up in our culture, we explain to them why we are doing this, to keep them on track. To keep you calm. It will protect you. The faith base.

P1

Migratory experiences and differences in the availability of support depending on visa status were also significant:

When I came from Turkey to here, one of the biggest surprises I have come across was the, how common suicide was among younger generations of Turkish people here. I was shocked, I lived in Turkey for 19 years, and I’d never even heard of someone who has heard of someone else’s suicide. Like, never even came across it from the third degree or fourth degree. Many here, I spoke to a family member of someone who was a victim to suicide. The mother told me about her son’s suicide, and I was so shocked. And then I heard the same story from multiple other people as well, and it just made me realize how widespread it was here, in younger generations of Turkish people.

YP1

In summary, sociocultural factors influenced the use and application of DMH tools, with culturally diverse end users describing how their culture informed their understanding of mental health and help seeking.

### Generational Differences in Mental Health and Digital Literacy

There were notable differences between the 2 generations across both study phases, particularly in terms of accessing mental health professionals, digital literacy, and whether participants were first- or second-generation migrants. Language barriers were identified, with a parent stating as follows:

That support is lacking. So you can imagine for some of these cultural communities, English might be a barrier, language might be a very big barrier.

P6

Parents (P6, P2, P8, and P3) preferred confiding in a close friend and face-to-face interactions over digital therapies. By contrast, young people (YP8, YP10, YP3, and P8) seemed more willing to share their mental health experiences. A young person stated as follows:

I know that like when I was a young teenager, going through my rebellious phase in an ethnic household where I didn’t feel like it was safe to open up to my parents, the online anonymous aspect was what made online like, Tumblr, a safe place for me to, I guess, voice my mental health concerns about myself.

YP5

Some parents also believed that young people were more mentally healthy and digitally literate:

I think teenagers today are really, because they do things like that in school now, they understand about self-care, they do mindfulness, they do all that sort of thing as teenagers now in high schools and things. I think that the word self-care is so synonymous now, everyone knows what that means. There’s depths of it, of course, but to a teenager even knowing, and they know how much time they spend, too much time on social media, they know they need to get more sleep, but you know, when they do all those things and they know they shouldn’t eat McDonald’s every day, they’re not silly, these kids are smart.

P8

As a first-generation migrant, a parent felt that it was important not to impose her own cultural rules on her Australian-born children:

Because they’re going to be Indian Australians, or Indian whatever it is. Deep down, they’re Indian, they grew up as Australian children. They’re more Australian than Indian sometimes. So we don’t, um, there’s no hard and fast rules. We can’t tell them, you have to get up at 5 o’clock and pray.

P2

The discrepancy between how young people perceived mental health challenges compared to their parents was regularly raised, with a young person elaborating as follows:

I think another aspect of like, immigrant parents on things about mental health, gender identity, sexuality that’s a big thing that, I know people, who are even single parents, who come from the immigrant background, they don’t understand what their young child is going through with gender identity and all that stuff, because it’s...it wasn’t really a thing when they were growing up, and, suddenly it’s become something now, and they don’t know how to deal with it.

YP10

In summary, generational differences were a central social factor that influenced the use and application of DMH tools.

### Stigma and Culturally Based Discrimination

Challenges with mental health stigma were discussed in both phases, with participants explaining that different cultural beliefs informed alternative explanations for poor mental health. Moreover, concerns were expressed that assigning a mental health diagnosis to a young person could lead to further marginalization because it might trigger defensive barriers in community members and contribute to increased ostracization. These experiences were centered on both internalized and externalized mental health stigma from family, friends, and extended cultural communities (P6, P3, P2, YP7, and YP3).

When discussing a potential mental health diagnosis for a child or young person, a parent stated as follows:

It’s denial first, thinking that nothing is wrong with your child...automatically you think, my child is fine, there’s nothing wrong...So the acceptance [of poor mental health] is going to be very difficult.

P2

For a young person with culturally diverse parents, the experience of mental ill-health was particularly challenging:

When my parents found out that I had some mental health issues...I noticed changes in their behaviors and in terms of my parents and carers being from an ethnic environment, they’re very judgmental. I just feel like my parents have zero understanding on mental health and mental illness so they wouldn’t take any of that as a form of “I need help.” They’ll take it as I’m being ungrateful.

YP7

A parent described how “mental illness ignites defensive barriers within people” (P6), suggestive of an internalized resistance that may impact help seeking in a community. A young person stated as follows:

It might also depend on the person and if they have internalized stigma. I think if an app has some sort of measure that can account for that [internalized stigma]. And that can frame the message depending on that, it would be really nice. So, even in ethnic communities, even if somebody is seeking help, for example. Learning that you’re depressed can be quite burdensome on your own mental health. The diagnosis might actually worsen your situation. Because the view of depressed people within your culture might always be these helpless people who cannot do anything basically.

YP1

Regarding the diagnostic potential of DMH tools, parents were cautious about making their children “feel like they’re different” (P8). A parent described how the diagnosis could be enduring and punitive:

You’re assuming the parent can put a diagnosis on a condition and that’s going to stick somehow. Because you’re sort of leading them down a particular path. I’m a great believer in diagnoses, but a diagnosis can be a sentence. Rather than a word, you know.

P7

There were concerns that a diagnosis can result in “lifelong stigma for a child or young person” (P7). A parent put it as follows:

You know what, suddenly my kid gets this thing as a mental health patients, that’s the end of their life. For the rest of their life they’ll be on medication or for the rest of their life they’ll be on this. They’re branded like that.

P2

There was a perception among some parents that they would “lose face” and be stigmatized in the eyes of their community if they or their child received a mental health diagnosis:

When we talk about the ethnic communities, for them, their saving face becomes a very big issue. And you know, none of them want to lose face in the community in public and what have you. If somebody else doesn’t even use their name then, for them, that stigma sort of stays, it’s always going to take me years to rebuild that trust again.

P6

Participants in both phases raised concerns about culturally based discrimination when seeking mental health support, including an inequity in the availability of psychological assessments, screenings, and support. Psychological assessments and mental health screenings were identified as a form of systemic discrimination by the young participants, who noted how most tools are only available in English, with questions reflecting Western understandings of mental health. This often results in people performing poorly due to an inability to understand the questions and being misdiagnosed. A young person described her lived experience of working in a Turkish-speaking psychologist’s office in Australia and raised her concern about the limited number of bilingual psychologists:

They were one of the few Turkish-speaking psychologists we had, four or five months of waitlists. So, it was cheaper than private practitioners. Only 20% of the fee would be paid out of pocket. And it was impossible to get through. Even if you have, um, some sort of government supplement like Medicare or some sort of private insurance, it was just impossible to make an appointment with her. And the same thing was the case for our Bosnian psychologist and our other psychologist as well.

YP1

Difficulties accessing mental health support were raised across both groups, with participants discussing the challenges, such as visa status, that migrants face when accessing mental health services, which can prevent them from receiving the same standard of support as other residents. This was particularly important for young international students waiting for permanent residency visas:

But because they’re still waiting for that process to get their PR [permanent residency visa]. They’re [asylum seekers] still in our schools. They go to private schools. They’re living a good life. But because they’re asylum seekers. We’re international students. We don’t have access to Medicare.

YP2

Discrimination from family members, friends, ethnic communities, and third-party systems upon receiving a mental health diagnosis was a concern raised by both groups (YP5, YP6, P6, and YP1). Both groups identified concerns about future discrimination from third parties who may use their mental health diagnoses to deny visa applications, steal their identities, or charge them higher insurance premiums (P1, P3, P5, P6, YP5, and YP6). In summary, stigma and diagnostic barriers, culturally based discrimination, and third-party access that may impact visa status and migration were central concerns.

## Discussion

### Principal Findings

The aim of the project was to explore the views of culturally diverse parents and young people on the opportunities and barriers to engagement with a web-based DMH screening tool. Through this work, we have explored the benefits, burdens, and trade-offs of using DMH tools from the perspectives of culturally diverse end users. On the basis of these findings, we provide our provisional set of reflections on the future of culturally sensitive DMH tools. Consideration of parental influence based on their country of origin, culture, and level of mental health literacy and how that is paired with raising a child in Australia is complex. Young people were perceived to have a higher level of general mental health and digital literacy, with greater awareness of their mood and self-care routines, as well as an understanding of healthy diet and lifestyle routines. They were also more willing to share information about their mental health with others. These generational differences highlight the importance of accounting for perceived barriers such as the influence of culture on mental health support, culturally based discrimination in seeking mental health support, and how a diagnostic label may impact the likelihood of engaging in help seeking.

### Leveraging Family Connectedness

We found that our participants’ understanding of their mental health was centered on their connection to family and parent-child relationships. Our findings suggest that family ties and family connectedness may influence the uptake of mental health treatments or interventions or whether and with whom DMH tool recommendations are shared. For culturally diverse youth, families provide an immediate social context (eg, as YP4 wrote in the emotional walk-through booklet: “The ‘Mental health’ term itself was still a taboo in India. We give importance to family connection and bonding [as a well-being strategy] but always have learnt to support and be there for one another”). Previous research found that the pace of cultural adaptation differs between parents and young people who resettle in Australia, with young people adapting faster but finding it hard to become independent from their families [16,88].

Family connectedness has been linked with lower odds of significant stress and despair for youth during acculturation and may protect them from other risk factors. In fact, the quality of family relationships can have a positive effect on well-being [89-91]. More research identifying positive family processes (eg, closeness with both mother and father) that build emotional well-being in culturally diverse young people is needed [92]. We found that familial influence informed young people’s understanding of mental health, which, according to some parents, could be explained and managed through religious practices or alternative methods. This is an important finding because it suggests that future iterations of DMH tools should emphasize the inclusion of culturally sensitive parental support and psychoeducation on mental health problems. It also highlights the importance of engaging community leaders, elders, religious leaders, or mental health advocates from different cultures and communities to gather their insights on and explanations of well-being and mental health to ensure that the technologies are culturally safe. We suggest that culturally appropriate and culturally informed psychoeducation for families is needed to encourage community buy-in, ultimately leading to more effective DMH tools for culturally diverse populations. This education would have to be offered thoughtfully, paired with data governance education to promote digital literacy and awareness of the insufficiently regulated data industry and unregulated practices that exist currently.

### Culturally Based Discrimination: Stigma, Loss of Face, and Collectivist Approaches to Mental Health

Barriers such as stigma, shame, and perceived judgment from community members were regularly raised. Many parents voiced concern about losing face in their community. Collectivist cultures that share a collective identity, emotional dependence, and shared duties and obligations operate under a “we” consciousness. These cultures are common outside Western societies that prioritize individualism [93]. The difference between individualism and collectivism ought to be considered when creating DMH tools, building an awareness that psychological support may be shared within a broad family network and that the “burden of results” may not be carried by the individual alone in collectivist communities. People from many collectivist cultures may struggle to understand the typical individualistic approach to treatment because they expect to receive care within the context of their family [94].

Some people may also have deeply held negative beliefs or attitudes toward those experiencing mental health challenges, making it important to design mental health services that incorporate informal support avenues (eg, friends and family) on which individuals may rely for help [95,96]. Loss of face is an important cultural factor in the Asian context and has been found to be a significant predictor of self-stigma and public stigma that impacts attitudes toward help seeking [97-99], especially given that community belonging plays a large role in migrant mental health [12].

Culturally diverse communities may minimize the reporting of psychological symptoms or may be resistant to sharing personal health information because of mistrust, perceived racism, or a sense that public mental health services do not accommodate or respect their cultural beliefs [100,101]. Culturally sensitive psychoeducation within communities is needed to combat stigma. To achieve this, we suggest engaging with community and religious leaders as well as mental health advocates with lived experience of mental health challenges, given their influential reach among diverse end users, to promote community-informed education, self-determination, and self-advocacy. DMH tool designs should offer transparent data collection policies, with tiered consent, to ensure that end users feel comfortable sharing their data, and this should be paired with the inclusion of digital navigators, who are members of health care teams dedicated to help end users [102].

Young people in our study voiced concern about the lack of culturally and linguistically diverse clinicians and assessment tools, noting that information was mostly communicated in English using a Western framework for assessing mental health. Research has found that diagnostic assessments tend to not be sensitive to racial and ethnic minority populations due to their exclusion from mental health research [103,104]. Furthermore, culturally diverse community members are less likely to have satisfactory access to mental health support and are less likely to receive a diagnosis for a mental health condition [105,106]. If DMH tools are to be relevant, accessible, and effective for culturally diverse populations, future designs and implementations must be available in languages other than English and incorporate cultural approaches to mental health. The very term *mental health* is a Western concept, and Western cultural traditions and understandings have informed much of the theory, practice, and understandings of mental health, including within psychology and psychiatry, with a central focus on individual pathology instead of sociocultural contexts and determinants [107,108]. Furthermore, how patients from culturally diverse backgrounds express their symptoms may vary, making it challenging to accurately diagnose or treat their conditions [104]. Individualized models of care may not be appropriate for those who are accustomed to community- or family-focused models of care [108]. The British Psychology Society proposed the power threat meaning framework as a nonmedicalized and nondiagnostic approach that instead describes how coping and survival mechanisms are adapted based on lived experience, previous threats, and social context, accounting for cultural differences in the experience and expression of distress, with less emphasis placed on Western views [109].

We ought to consider how to design culturally sensitive DMH tools that are informed by attitudes from diverse communities and encourage self-determination and cultural context within a well-being framework. One suggestion is for DMH tool developers to offer enhanced psychoeducation that is culturally safe and fosters self-determination, with tailored psychological assessments and resources that account for language variations and barriers for users coming from collectivist cultures whose conceptions of mental health vary from the Western medicalized understanding.

### Medicalization by Design

Concerns were raised about making a child or young person feel different. Participants worried that “a diagnosis may worsen your situation,” suggesting that a diagnostic label or the detection of a mental health problem, as well as the diagnostic process itself, may further marginalize diverse communities through the medicalization of culturally appropriate cognitions or behaviors. Medicalization pathologizes behaviors according to a Western psychiatric framework, putting the responsibility back on the individual to stay healthy without considering other important social factors [110,111]. Online screening tools and mobile apps are being designed to promote well-being and provide psychological support, but they can also work to endorse ongoing surveillance [112] of mood and activity and set expectations about healthy behaviors and cognitions. A shift is needed in how mental health is conceptualized and designed for in a way that accounts for cultural barriers that may limit engagement and usability for diverse end users of DMH tools [109]. Importantly, we need to invest in community-led practices that promote community leadership in the design and innovation of DMH tools [82] to avoid built-in discrimination that reproduces inequities due to normative or Western-oriented assumptions about mental health [113]. We suggest that when designing DMH tool outputs, such as the communication of health information, recommendations for services and interventions, and referral pathways, the reported symptoms entered by the end user are phrased in a way that avoids pathologization and diagnosis. Currently, DMH platforms tend to recommend resources on anxiety, depression and suicidal ideation, and eating disorders [39,114]; however, we believe that it is imperative that symptoms are spoken about in clusters, rather than specifying specific diagnostic criteria that may then pigeonhole or “label” an end user without appropriate access to clinical care.

### Attitudes Toward Data Collection Are a Two-Way Street

When using DMH tools, participants felt that there was less concern about privacy and data sharing when the data collected were more generalized. This is consistent with a recent study on diverse communities using mindLAMP, a DMH platform, where participants wanted their DMH apps to pull data from their current health care records to enhance app personalization [115]. Participants acknowledged that innovations and interventions require consent for collecting substantial amounts of data to tailor services or for platforms to “algorithmize my personal experiences”; however, research has found that DMH tools are often not culturally tailored or responsive due to culturally diverse or racial and ethnic minority populations often not being considered in their development and evaluation [116,117]. Young people from culturally diverse communities recognized that they would need to provide access to personal digital health data to realize the benefits promoted by DMH tools. Critics have argued that young people may not comprehend the longevity and potential harms of a digital footprint and that thoughtful education and support around this is important for their future privacy [118]. While research has demonstrated an unwillingness from end users to share their sensitive health data with commercial organizations [23,119-121], there was a sense in our findings that participants expected an “algorithmized experience,” which would require health data to be treated as open data [122]. Due to the sensitive nature of mental health data, further education on data governance and the potential reuse of big data is recommended for young people, given the nature of cross-sectoral data sharing. One approach may be incorporating speculative design approaches, such as the one suggested in the study by McNaney et al [23], to engage young people in discussions around their digital futures, the potential harms of data sharing, and the misuse of their data.

In both phases, the participants identified concerns about data breaches, including potential identity theft and the impact it could have on visa and migration status. These concerns are reflected in the literature, with 1 study finding that nearly half of the DMH apps surveyed did not have a privacy policy, although it is known that health care apps often share data points (eg, age, contact information, and other user data) with multiple third parties for commercial purposes [123,124]. Efforts have been made to establish ethical standards for digital data gathered from DMH tools, including accounting for data breaches, which advise developers to account for hidden assumptions and unintended consequences [125]. Regulations such as the General Data Protection Regulation in Europe and similar privacy acts being developed in Brazil, Chile, and Canada aim to protect data privacy. However, even the General Data Protection Regulation has limitations, particularly in the context of emerging generative artificial intelligence innovations.

### Reflections and Limitations

This study sought to engage with culturally diverse representatives in relation to cultural background and lived experience of mental health challenges. To achieve this, we approached an organization focused on multicultural mental health that advertised the study, recruited participants, and supplied bicultural workers for our study. While we were able to have meaningful discussions in our workshops, the participants were all affiliated with the organization, meaning that the sample was not necessarily representative of a truly culturally diverse population, given that each participant was already mental health literate, able to communicate in English, and held a preconceived understanding of mental health, rather than being a layperson. The addition of an Anglo-Australian participant was also a limitation. Future research needs to engage with a broader range of culturally diverse participants, including those with less digital and mental health literacy and those who may be in a more contemplative stage of their mental health journey and possibly unaware of support services. Future research might consider recruiting through youth organizations or well-being counselors who may have access to a more culturally diverse range of participants who reflect a more culturally diverse picture of mental health literacy and help seeking. Future research should also engage in participatory action research methods (eg, co-design and coproduction) that allow end users to develop RQs and study designs. However, this approach can present additional challenges to building social capital and gaining trust to engage with culturally diverse end users in a meaningful way, and this needs to be factored into study timelines. Our study was bound to an innovation timeline, making the recruitment of a truly diverse sample impossible. We were conscious that we did not invite the participants to review the data analysis or final manuscript, which could have reduced the power imbalances further by allowing the participants to join as coresearchers in the project from start to finish. Future iterations would benefit from inviting culturally diverse participants on board as coresearchers, not just as participants. We believe that it is important to embed the voices of diverse community members within a research project throughout its lifespan.

Most of our participants were from South-East Asia. While this is an accurate reflection of the majority of culturally diverse Australians [1], it may not capture the views of ethnic and racial minority cultures within Australia. We hoped to avoid essentializing all experiences as one by including quotes from individuals because we believe that culturally diverse communities do not share a single common experience due to varying sociopolitical differences such as age, culture, and citizenship. The median age of the young participants was relatively high, with one aged 16 years and one aged 18 years, while the remaining participants were aged 20 to 22 years. As such, our findings may not reflect the views of younger adolescents. Future endeavors should aim to apply the insights gained from this research in the development and pilot testing of DMH interventions and applications by evaluating their effectiveness and refining them based on the outcomes.

### Conclusions

Inclusive design considerations for the future development of DMH tools that account for culturally based discrimination, are culturally safe, consider family and cultural influence on mental health support, and are thoughtful in how mental health problems are detected and communicated to end users would improve engagement in DMH tools and help seeking. We recommend encouraging culturally sensitive psychoeducation, thoughtful nonpathologizing language that avoids providing a specific diagnosis, and the inclusive involvement of community leaders and mental health advocates in the future design of DMH tools to ensure that they are designed to meet the needs of culturally diverse end users.

## Appendix

Multimedia Appendix 1 Digital mental health co-design workshop schedule.

## Abbreviations

DMH: digital mental health
GMCI: Growing Minds Check-In
GP: general practitioner
RQ: research question
