# Supplementary material for: Developing Digital Mental Health Tools With Culturally Diverse Parents and Young People: Qualitative User-Centered Design Study
Source: JMIR Pediatr Parent. 2025 Apr 22;8:e65163. doi: 10.2196/65163 (PMC12056437; doi:10.2196/65163)
Supplement: Multimedia Appendix 1 [file pediatrics_v8i1e65163_app1.docx]

Appendices

### Digital Mental Health Co-design Workshop Schedule

**Workshop aims:**

- To identify the possible benefits, burdens, or trade-offs of how mental health data is collected, shared, and used.
- To examine how end-users value data about mental health and whom they will share their data with.
- To identify the social factors that influence the use and application of digital technologies, including third-party use.
- To examine how predictive information is communicated and understood, and to

what impact on clinical practice this might have.

| Duration | Exercise | Resources |
| --- | --- | --- |
| 10am | Workshop Commencement |  |
| 10 mins | Welcome and introduction to the project  **S**: explain session safety and etiquette  **I**: explain overarching purpose of study  Prompts:   - Consent required? If so, sign forms - Cover study aims and workshop aims (slides) - In each of the activities we invite you to imagine yourself as a future user of digital mental health technology, it can be speculative and you don’t have to discuss personal anecdotes if you don’t feel safe to do so | - Consent forms - Information sheets - Pens - Audio recorders |
| 10 mins | **Persona Icebreaker:**  **S:**  What is your understanding of mental health? What makes for good mental health? What makes for bad mental health? What can influence it?  **I**: write down suggestions on sticky notes  Group discussion to set the context for the following activities. This activity will help to get to know how participants understand mental health. Done via informal chat using a whiteboard to get to know different members of the group.  **Aim:**  To examine how end-users from priority groups perceive mental health and whom they will share their data with.  **Themes:**  mental health literacy and cultural fluency, social factors surrounding mental health | - Whiteboard - Marker pens - Flip chart paper |
| 30 mins | **Digital Vault:**  **Aim:**  This activity is designed to gauge participants' level of understanding of MH data, how secure it is, different types of data, and who might share or access it.  **Themes:**  Understanding of MH data, privacy, access  Instructions**:**   1. A physical box (representing the *digital vault - this vault will have 3 layers - the outer layer, an inner layer, and a chained safe*) will be placed at the centre of the room. 2. Set of pre-filled cards containing different forms of mental health data (e.g. social media apps, phone, diary, web-browser history) and blank ones that participants can write on to will be provided to each participant. 3. Ask participants to slot in the mental health data cards into specific areas of the vault they deem appropriate. 4. Probe why.   **I**: read script  **S**: help with arranging icons and keys within the vault, observing zoom participant and probing why  Script:  We will create a physical box to work as a vault. This digital vault or digital bank can store all of your mental health data safely. There is an outer vault, an inner vault, and a locked-up safe to contain important information about your mental health:   - social medial interactions - the apps on your phone - your internet browser history - the tone of your voice from phone calls you’ve made   What would you put in there? Why? What might you put right at the back of the vault, where it needs a key before it can be accessed? What don’t you mind putting at the front where it might be somewhat visible?  How do you feel about these data points passively tracking you? Why? | - Large sheets of paper - Pens - Post-it notes - Stimuli: pictures of potential mental health data e.g. icons of social media apps, phone, diary, etc. - Cardboard box and piping - Set of cards, some prefilled some blank |
| 30 mins | **Blank Keys:**  **Aim**:  This activity is designed to understand to whom the participants are willing to share their data with and their reasons why.  **Themes**:  understanding of MH data, privacy, access  **Instructions:**   1. Give participants 6 key to the digital vault (i.e. post-it notes / key-shaped cut-outs); 2 keys for the inner vault, 2 keys for outer vault, 2 keys for the safe. 2. Ask the participants to write on to these blank keys the people they are willing to give access to the different areas of the digital vault. 3. Probe why. 4. Read the scenario about digital bank robbers and ask the probing questions.   **I:** read script  **S:**  observe comments, probe and build on what participants raise  **Script**:  Who would you give the keys to the vault to? Your family, GP, spiritual leader, friends? Insert the keys into the vault in a position either on the inner or outer part of the vault depending how much you trust that person with the key. Why have you chosen this person? Why are they important to you? (15mins)  Now let's imagine digital bank robbers broke into your vault. Who might the robbers be? Why would they want your data? Which specific area of the vault would they be most interested in? (15 mins) | - Key cut outs - Pictures of robbers |
|  | **Leg Stretch Break 10 min** |  |
| 45 mins | **Data Sharing**  **Aim**:  This activity is designed to get participants thinking about their individual perspectives as well as how others may feel about having their personal data shared under the guise of helping them or the community.  To identify the social factors that influence the use and application of digital technologies, including third-party use and sharing.    **Themes:**  Data sharing, privacy, and control  **Instructions:**   1. For Sub activity 2: This is just a discussion based activity. Read the scenario and answer the probing questions.   **I:** read script  **S:**  observe comments, probe and build on what participants raise  **Script**:  ***Sub-activity 1: YOU AS THE OWNER OF THE PREDICTION (25 mins)***  Imagine your digital vault is now managed by a digital manager. This digital manager has worked out how to use the data points that were passively collected in your vault to predict mental health issues. The manager has indicated you are likely to have complex mental health (bi polar or schizophrenia).  Your digital vault has come back with a diagnosis/prediction you may have complex mental health condition. Would you want anyone to know about what was predicted from the digital vault? Who do you think the manager should share this information with? Who wouldn’t they share this with?   1. Family:  - Do you think the manager should notify your partner or close friends? Why? Why not?  1. University:  - How would you feel if the university knew about your digital vault prediction? Who would you feel safe sharing this with at university? What would you be worried about?  1. Current / Potential Employer:  - How would you feel if your employer, knew about your digital vault prediction? Who would you tell? What would you be worried about?   ***Sub-activity 2: YOU AS A RECIPIENT OF OTHERS’ PREDICTION (20 mins)***  Imagine the vault manager has shared with you that your friend or colleague has a concerning outcome in their mental health prediction based on their digital vault data.  What would you do with this information? Who would you share this with? Who wouldn’t you share this with? | - Pen - Post-it note - Picture prompts |
|  | **Lunch Break 15 mins** | Lunch |
| 30 mins | **Future forecasting return of results data sharing:**  **Aim**: To examine how predictive information is communicated and understood, and to  what impact on clinical practice this might have. This is a discussion-based activity to consider the modalities data donated today could be used in the future and whether digital mental health literacy and MH data counsellors are efficient.  **Themes**: Privacy, safety, trust, informed consent, risk communication (triage officer), inherent uncertainty  **Instructions:**   1. Co-facilitator to read the script about imagined future. 2. End with a discussion around information presentation of mental health predictions (sub-activity 2)   **I:** read script  **S:**  observe comments, probe and build on what participants raise  **Script:**  Imagine that in 20 years from now the uploads from your cloud storage have been combed continuously for analysis. Scientists have worked out a way to use your old cloud entries to predict possible signs of Parkinson’s based on your data engagement, years and years before they are usually diagnosed. You’re sipping a coffee at work one day when you receive an alert on your smart device. It’s notifying you that there are important results in your digital inbox. You had forgotten about participating in this study and what you’d agreed to let the digital vault have secured, but the alert indicates it’s about findings from a research study in 2022. The alert is a prediction that you’re likely to have early-onset Parkinson’s based on the data stored in your digital vault.  I: read script  ***Sub-activity 1: Emojis (15 mins)***  How do you feel about getting this result? [Anxious, relieved, glad, etc.].  Would you like to know this in advance? (  Do you feel empowered knowing this prediction in advance? Do you trust its accuracy?  Would you prefer your GP or mental health clinician had told you? Would you trust it more if your GP told you? Are you concerned about who else is accessing this digital vault from some years ago? (20 min)  ***Sub-activity 2: Discussion (15 mins)***  ***Sarah:*** *read script*  How would you like this prediction presented to you? How would this information be presented back to you in a way that you understand and is meaningful? | - Post its - Pens or markers - Emoji icon cut outs |
| 5 mins | Wrap up and goodbye |  |
